# Treatment patterns and outcomes of patients with metastatic non-small cell lung cancer in five European countries: a real-world evidence survey

**DOI:** 10.1186/s12885-023-11074-z

**Published:** 2023-06-30

**Authors:** Hollie Bailey, Adam Lee, Laura Eccles, Yong Yuan, Helen Burlison, Cameron Forshaw, Nebibe Varol

**Affiliations:** 1 Adelphi Real World, Adelphi Mill, Grimshaw Lane, Bollington, Macclesfield, Cheshire, SK10 5JB UK; 2 Bristol Myers Squibb, Uxbridge, UK; 3 Bristol Myers Squibb, Princeton, NJ USA

**Keywords:** Europe, Metastatic, Non-small cell lung cancer, EGFR wild-type, ALK wild-type, Real-world, Survey, Treatment, Treatment outcomes

## Abstract

**Background:**

The landscape of non-small cell lung cancer (NSCLC) therapy is rapidly changing. This analysis aimed to understand patient characteristics, diagnosis and treatment patterns in patients with metastatic NSCLC (mNSCLC) without *EGFR* and *ALK* mutations across five European countries.

**Methods:**

Data were drawn from the Adelphi NSCLC Disease Specific Programme™, a point-in-time survey of oncologists/pulmonologists and their consulting patients in France, Germany, Italy, Spain and UK. Physicians completed record forms (RFs) for the next six consecutive consulting patients with advanced NSCLC, who then voluntarily completed questionnaires. As an oversample, physicians provided a further ten RFs specifically for patients with *EGFR*-wild-type mNSCLC: five patients diagnosed before March 2020 (pre-SARS-CoV-2 [COVID-19]) and five patients diagnosed from March 2020 (during COVID-19). Only *EGFR*-wild-type/*ALK*-wild-type patients were included for analysis.

**Results:**

Mean (standard deviation [SD]) age for 1073 patients with *EGFR*-wild-type/*ALK*-wild-type mNSCLC was 66.2 (8.9) years, 65.2% were male and 63.7% had adenocarcinoma. Level of PD-L1 expression at advanced diagnosis was < 1% for 23.1% of patients, 1–49% for 40.9% and ≥ 50% for 36.0%. Most common first-line (1L) advanced treatment was chemotherapy only (36.9%), immunotherapy monotherapy (30.5%) or immunotherapy + chemotherapy (27.6%). Of 158 patients who had progressed beyond 1L therapy, the mean (SD) time-to-treatment discontinuation was 5.1 (4.3) months; 75.9% of whom completed their 1L treatment as intended. A complete response was achieved by 6.7% and a partial response by 69.2% of patients. Of 38 patients who discontinued 1L treatment early, disease progression was reported for 73.7%. Quality of life (QoL) reported by patients was generally lower than normative reference values. Of 2373 oversample patients, physicians reported management changes for 34.7% due to COVID-19, ranging from 19.6% in Germany to 79.7% in the UK. Immunotherapy was prescribed as 1L NSCLC treatment during COVID-19 for 64.2% (*n* = 786) of patients and pre-COVID-19, for 47.8% (*n* = 549).

**Conclusions:**

Real-world treatment patterns suggest that chemotherapy use remains high despite guidelines recommending immunotherapy-based 1L treatment for mNSCLC. QoL reported by patients was generally lower than population reference values. Not implying causality, 1L immunotherapy use was higher during COVID-19 than pre-COVID-19, and the UK saw the biggest impact to patient management due to COVID-19.

**Supplementary Information:**

The online version contains supplementary material available at 10.1186/s12885-023-11074-z.

## Introduction

Lung cancer is the leading cause of cancer deaths globally, accounting for 18% (1.8 million) of deaths and 11.4% (2.2 million) of new cases in 2020 [1, 2]. In Europe alone, the prevalence, incidence, and death rates in 2020 were 22.4%, 21.6% and 21.4%, respectively [1]. Non-small cell lung cancer (NSCLC) accounts for about 85% of all lung cancer cases [3, 4], with initial diagnosis most commonly in the advanced stages [5]. The overall relative 5-year survival rate for lung cancer is 23%, although this varies depending on clinical stage, and is 61% for localised, 34% for regional, and 7% for metastatic disease [5]. Diagnosis at an advanced disease stage means that the majority of lung cancers are ineligible for potentially curative surgery, unlike in the non-metastatic disease stage.

Alongside clinical understanding of the malignancy, the treatment landscape for metastatic NSCLC (mNSCLC) has evolved considerably over the past few decades; first with cytotoxic chemotherapy (from 2006), followed by targeted therapies (mainly from 2011) for patients with oncogenic driver mutations and, more recently, immunotherapy (from 2015) for patients without actionable mutations [6]. Most NSCLC tumours in Europe lack oncogenic driver mutations, rendering patients without targetable mutations ineligible to receive targeted therapies [7]. Additionally, most oncogene-driven NSCLC tumours initially responding to targeted therapies such as epidermal growth factor receptor (EGFR)-tyrosine kinase inhibitors (TKI) and anaplastic lymphoma kinase (ALK) inhibitors eventually progress over time as they acquire drug resistance [8, 9].

The identification of mutations in the *EGFR* gene (*EGFR*-mutant [*EGFR*-mut]; coding for a receptor tyrosine kinase) and rearrangements in the *ALK*gene, found primarily in tumours of non-squamous histology, has led to the development of targeted therapies, EGFR-TKIs (e.g., erlotinib, gefitinib, afatinib, osimertinib, and dacomitinib) and ALK inhibitors (e.g., crizotinib, alectinib, ceritinib, brigatinib and lorlatinib), respectively, for patients with advanced non-squamous NSCLC [10, 11]. The discovery of targetable mutations in genes other than *EGFR* and *ALK*, such as c-ros oncogene 1 (*ROS1*) and v-Raf murine sarcoma viral oncogene homolog B (*BRAF*) has led to the development of additional targeted therapies in NSCLC, specific to each genomic mutation [11].

Immunotherapies have also been developed to treat patients with NSCLC and have shown that they may be an effective 1L treatment for patients whose tumours do not harbour oncogenic driver alterations. Programmed death-1 (PD-1) is an inhibitory T cell receptor that, when bound to its ligands PD-L1 and PD-L2, induces inhibitory messaging leading to a reduction in T-cell proliferation, cytokine production, and cytotoxic activity [12, 13], thus acting as an immunologic checkpoint. PD-L1 expression occurs in many different tumour types including lung [14, 15]. PD-1 expression on lymphocytes and its interaction with its ligands on tumour and immune cells are the basis of anti-tumour immunity and PD-1 inhibition in cancer immunotherapy [16]. For patients without oncogenic driver mutations, treatment options can be considered based on PD-L1 status [11]; immunotherapy as a monotherapy can be used for patients with tumour PD-L1 ≥ 50% and immunotherapies can be used in combination with chemotherapy in patients irrespective of PD-L1 status but mostly preferred for those with PD-L1 < 50%.

An understanding of real-world treatment patterns and outcomes can provide important context for the rapidly changing landscape of NSCLC therapy, whilst further contributing to determining the applicability of clinical trial evidence to the real-life clinical setting where patient populations are more diverse and typically have more comorbidities. The primary objectives of this analysis were to understand patient characteristics, the diagnostic landscape, and treatment patterns particularly in the first-line (1L) setting in patients with mNSCLC without *EGFR* and *ALK* mutations across Europe. Further objectives were to evaluate the burden of illness and unmet needs in patients with *EGFR*-wild type (WT)/*ALK-*WT mNSCLC in the 1L setting. The impact of SARS-CoV-2 (COVID-19) on 1L diagnostic and treatment patterns in patients with mNSCLC without *EGFR* and *ALK* mutations was also explored.

## Methods

### Survey design

Data were drawn from the Adelphi NSCLC Disease Specific Programme (DSP)™, a multinational, point-in-time survey of physicians and their patients. Data were collected for the main sample from July 2020 to November 2020. A retrospective oversample was also conducted as part of this DSP from May 2021 to August 2021.

The DSP methodology has been previously published and validated [17–20], with studies across many different disease areas implemented globally. The survey included a physician survey and workload questionnaire, a physician-reported electronic patient record form, and a voluntary patient-reported questionnaire. Physicians and their patients were recruited from five European countries (France, Germany, Italy, Spain, and the United Kingdom [UK]).

### Survey population

For the main sample, physicians (oncologists/pulmonologists) were included in the study if they were actively involved in the management and systemic treatment of patients with mNSCLC and consulted at least three patients with mNSCLC in a typical month. Patients aged 18 years or over with a physician-confirmed diagnosis of mNSCLC (stage IIIb–IV) and not part of any clinical trial were eligible for inclusion in the main sample analysis.

For the oversample, oncologists/pulmonologists were included in the study if they were actively involved in the management and systemic treatment of patients with *EGFR*-WT mNSCLC and had a clinical workload of at least five patients with *EGFR*-WT mNSCLC (recurrent or de novo) diagnosed between March 2020 (a date where all five European countries were in lockdown due to COVID-19) up to when data collection ended (August 2021), and at least five patients with *EGFR*-WT mNSCLC diagnosed in the six months prior to March 2020. Patients aged 18 years or over with a physician-confirmed diagnosis of *EGFR*-WT mNSCLC and not part of any clinical trial were eligible for inclusion in the retrospective oversample.

### Participant selection and data collection

Patients in the analyses include two cohorts, randomly sampled patients (the main patient sample) and an additional retrospectively captured set of patients (oversample). The main sample focused on mNSCLC, providing data to reflect current clinical practice at the time of survey. For the main sample, a geographically representative sample of oncologists and pulmonologists were recruited. Physicians meeting the inclusion criteria and willing to participate first completed an attitudinal survey regarding the management and treatment of patients with mNSCLC. Physicians were then asked to complete a patient record form for their next six consulting patients with mNSCLC who met the patient eligibility criteria. As patients consult at random, the patient sampling method is considered to generate a patient sample representative of the typical mNSCLC consulting population.

For the oversample, physicians provided information retrospectively on 10 patients with *EGFR*-WT mNSCLC: five patients diagnosed during the pre-COVID-19 period (defined as 1 September 2019 to 29 February 2020; prior to when all five European countries went into lockdown due to the COVID-19 pandemic) and five patients diagnosed during the COVID-19 pandemic (1 March 2020 to the time of data collection). This was in order to investigate the effects of COVID-19 and ‘lockdown’ on the treatment and management of mNSCLC.

For both samples, physicians completed an electronic patient record form for each patient who met the inclusion criteria, with data extracted from patient medical records. Data included patient demographics and clinical characteristics, diagnostic tests/assessments, biomarker status at advanced stage diagnosis, prior treatment history and associated outcomes, healthcare resource use (HCRU), and hospitalisations.

For the main sample, physicians invited the same patients for whom they completed an electronic patient record form to complete a voluntary patient-reported questionnaire. As the oversample was retrospective, these patients did not complete these questionnaires. The patient-reported questionnaire collected data on patient demographics, disease burden, and quality of life (QoL). QoL was measured using the EQ-5D-5L [21, 22], and the Functional Assessment of Cancer Therapy (FACT), including the FACT-General (FACT-G) and FACT-Lung (FACT-L) [23, 24]. The EQ-5D-5L French value set was used for all countries to remove bias in cross-country comparisons due to country differences in value sets [25].

Using a checkbox, patients provided informed consent to take part in the survey. Data were collected in such a way that patients and physicians could not be identified directly. Physician and patient data were pseudo-anonymized. A code was assigned when data were collected. Upon receipt by Adelphi Real World, data were pseudo-anonymized again to mitigate against tracing them back to the individual. Data were aggregated before being shared with the subscriber and/or for publication.

Data collection was undertaken in line with European Pharmaceutical Marketing Research Association guidelines [26] and as such it did not require ethics committee approval. Each survey was performed in full accordance with relevant legislation at the time of data collection, including the US Health Insurance Portability and Accountability Act 1996 [27], and Health Information Technology for Economic and Clinical Health Act legislation [28].

### Analysis

**Fig. 1:**
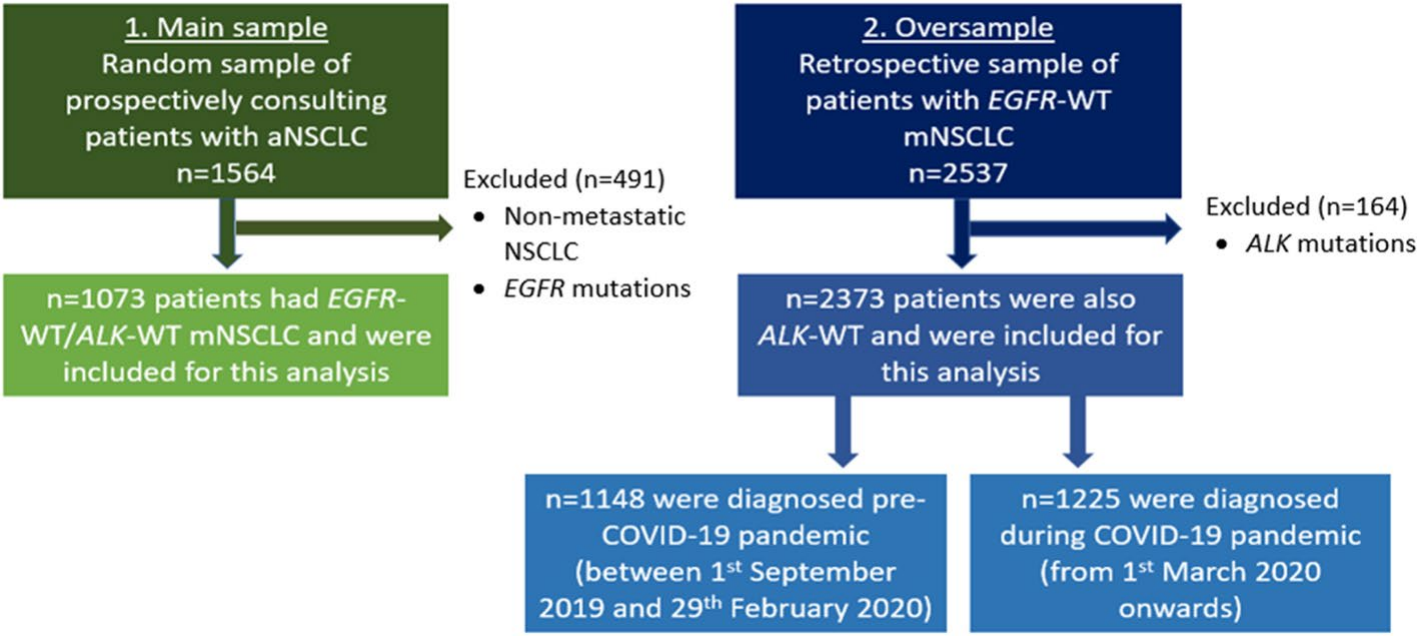
Patient selection and sample sizes. Legend. ALK, anaplastic lymphoma kinase; aNSCLC, advanced non-small cell lung cancer; COVID-19, SARS-CoV-2; EGFR, epidermal growth factor receptor; EGFR-WT/ALK-WT, (i.e., no sensitising EGFR mutation or ALK translocation; wild type); mNSCLC, metastatic non-small cell lung cancer

Analyses were performed separately on the main sample and the retrospective oversample. Main sample data were analysed as aggregated values and by country (France, Germany, Italy, Spain, UK). They were also stratified by line of therapy (1L only presented), by biomarker status (PD-L1 expression ≥ 50%, 1*–*49% and < 1%), by 1L mNSCLC treatment, and by *EGFR* and *ALK* biomarker status (only *EGFR*-WT/*ALK*-WT patients are included in this analysis). Analyses of the retrospective oversample were stratified by patients diagnosed pre-COVID-19 (up to six months prior to March 2020) and during the COVID-19 pandemic (from March 2020 to time of data collection) (patients with tumour *ALK* mutations were excluded from this analysis). Patient selection and sample sizes are shown in Fig. 1.

Data were summarized using descriptive analyses. Means and standard deviations (SD) were calculated for continuous variables, and frequency and percentages were calculated for categorical variables. Continuous variables were compared using t-tests or Mann–Whitney tests, depending on the distribution. Categorical variables were compared using Fisher’s exact tests for variables with two categories and Chi square tests for variables with more than two categories. Ordinal categorical variables were compared using Mann–Whitney tests. A *p*-value of less than 0.05 was taken as indicating between-group statistically significant differences. All analyses were performed using the software package IBM SPSS Data Collection Survey Reporter Version 7.5 and STATA® Version 16 (StataCorp LP, College Station, USA).

The EQ-5D-5L utility index assessed health status with regard to mobility, self-care, usual activities, pain/discomfort, and anxiety/depression. The EQ-5D visual analogue score (VAS) ranges from 0 to 100, where higher scores indicate better quality of life (QoL) and the EQ-5D-5L index total score (French value set) and domain scores range from 0.00 to 1.00, where higher scores indicate better QoL [21, 22] Data for EQ-5D-5L and EQ-5D VAS were compared with normative reference values (EQ-5D, France-specific time to trade off value set 0.892; VAS, overall mean of the total for the five European countries, 77.8) [25]. The minimal clinically important differences (MCID) for (UK based) EQ-5D utility index and EQ-5D VAS are 0.082 and 0.07, respectively [29, 30].

The FACT-G is designed to measure the physical, social, emotional, and functional well-being domains of QoL in patients with cancer [24, 31]. The FACT-G serves as a foundation upon which questions are added to address specific concerns or problems, e.g., to lung cancer FACT-Lung (FACT-L) with its lung cancer subscale and trial outcomes index. The ranges of possible total scores are 0–108 in FACT-G and 0–136 in FACT-L, with higher scores corresponding to a better QoL. Data for FACT-G were compared with normative reference values [32]. The MCID for FACT-L for advanced NSCLC is 2–3-point difference on the lung cancer subscale [33].

## Results

### Main sample

#### Physician-reporting and patient-reporting populations

From the Adelphi NSCLC DSP, 248 oncologists/pulmonologists (France: *n* = 48, Germany: *n* = 50, Italy: *n* = 50, Spain: *n* = 50, UK: *n* = 50) provided data for a total of 1564 eligible patients with mNSCLC and a total of 598 matched patients also completed the voluntary patient-reported questionnaire (Fig. 1). For this analysis, 1073 patients with *EGFR*-WT/*ALK*-WT mNSCLC were included (France: *n* = 264 [24.6%], Germany: *n* = 152 [14.2%], Italy: *n* = 201 [18.7%], Spain: *n* = 226 [21.1%], UK *n* = 230 [21.4%]). Patient-reported questionnaires were completed by 262 matched patients (France: *n* = 41 [15.6%], Germany: *n* = 57 [21.8%], Italy: *n* = 46 [17.6%], Spain: *n* = 83 [31.7%], UK: *n* = 35 [13.4%]).

#### Patient demographics and clinical characteristics

Patient characteristics for the total mNSCLC sample who were *EGFR*-WT/*ALK*-WT (*n* = 1073) are shown in (Table 1). At the time of data collection, patients’ mean age (SD) was 66.2 (8.9) years and 65.1% were male.

**Table 1:** Demographics and clinical characteristics of patients with *EGFR*-WT/*ALK*-WT mNSCLC

	**Total population**	**1L treatment at data collection**
Age, years		
n	1073	915
Mean (SD)	66.2 (8.9)	66.2 (9.0)
Sex, n (%)		
n	1073	915
Male	699 (65.1)	587 (64.2)
Female	374 (34.9)	328 (35.8)
Current disease stage, n (%)		
n	1073	915
Stage IVa	327 (30.5)	287 (31.4)
Stage IVb	746 (69.5)	628 (68.6)
NSCLC histology, n (%)		
n	1073	915
Adenocarcinoma	684 (63.7)	578 (63.2)
Squamous cell carcinoma	355 (33.1)	309 (33.8)
Large cell carcinoma	23 (2.1)	19 (2.1)
Other	11 (1.0)	9 (1.0)
Primary site of metastases (> 5%), n (%)		
n	1073	915
Contralateral lung	506 (47.2)	422 (46.1)
Lymphatic system/lymph nodes (any)	365 (34.0)	305 (33.3)
Bone	357 (33.3)	311 (34.0)
Liver	254 (23.7)	228 (24.9)
Pleura	257 (24.0)	213 (23.3)
Adrenal glands	224 (20.9)	191 (20.9)
Brain	80 (7.5)	65 (7.1)
Current ECOG PS score, n (%)		
n	1071	913
0	237 (22.1)	210 (23.0)
1	638 (59.6)	545 (59.7)
2	148 (13.8)	116 (12.7)
3	26 (2.4)	24 (2.6)
4	22 (2.1)	18 (2.0)
Current symptoms (most frequent 10), n (%)		
n	1073	915
Cough	636 (59.3)	547 (59.8)
Fatigue	473 (44.1)	387 (42.3)
Dyspnoea/breathing complications	454 (42.3)	390 (42.6)
Weight loss	299 (27.9)	248 (27.1)
Chest pain	273 (25.4)	235 (25.7)
Loss of appetite	284 (26.5)	240 (26.2)
Bone pain	262 (24.4)	222 (24.3)
Persistent cough	220 (20.5)	192 (21.0)
Anxiety	157 (14.6)	129 (14.1)
Low mood	172 (16.0)	142 (15.5)
Current comorbidities (> 5%), n (%)		
n	1073	915
Hypertension	399 (37.2)	344 (37.6)
Chronic pulmonary disease	256 (23.9)	217 (23.7)
Dyslipidaemia	163 (15.2)	135 (14.8)
Diabetes without chronic complications	130 (12.1)	113 (12.3)
Peripheral vascular disease	54 (5.0)	44 (4.8)
Current Charlson comorbidity index		
n	1073	915
Mean (SD)	0.5 (0.9)	0.5 (0.9)

*ALK* Anaplastic lymphoma kinase, *ECOG PS* Eastern Cooperative Oncology Group performance status, *EGFR* Epidermal growth factor receptor, *EGFR-WT/ALK-WT, i.e.*, no sensitising EGFR mutation or ALK translocation; wild type, *mNSCLC* Metastatic non-small cell lung cancer, *SD* Standard deviation, *1L* First-line treatment

Current: time of consultation

ECOG performance status scale: 0, Fully active, able to carry on all pre-disease performance without restriction; 1, Restricted in physically strenuous activity but ambulatory and able to carry out work of a light or sedentary nature, e.g., light house work, office work; 2, Ambulatory and capable of all selfcare but unable to carry out any work activities; up and about more than 50% of waking hours; 3, Capable of only limited selfcare; confined to bed or chair more than 50% of waking hours; 4, Completely disabled; cannot carry on any selfcare; totally confined to bed or chair; 5, Dead

Charlson comorbidity index: index ranges from 0, low risk of mortality attributable to comorbid disease, to ≥ 5, with stepwise increases in the cumulative mortality attributable to comorbid disease

At data collection, 30.5% and 69.5% of patients had stage IVa and IVb mNSCLC, respectively. Adenocarcinoma (63.7%) and squamous cell carcinoma (33.1%) were the most common histological types. Patients most frequently had metastases to the contralateral lung (47.2%).

Patients most frequently presented with cough (59.3%), fatigue (44.1%), and dyspnoea (42.3%). Hypertension and chronic pulmonary disease were the most frequently cited comorbid conditions experienced by patients with mNSCLC. The majority of patients had a current Eastern Cooperative Oncology Group performance status (ECOG PS) score of 1 (59.6%).

Of the 1073 patients that had *EGFR*-WT/*ALK*-WT mNSCLC, the majority had undergone a biopsy (89.0%), blood tests (85.7%), a computerized tomography (CT) scan (83.6%) and fluorodeoxyglucose*-*positron emission tomography (57.7%) to aid mNSCLC diagnosis (Supplementary Table 1). Tests that were used most frequently to aid diagnosis were also used most commonly for disease monitoring.

Overall, 1021 patients were tested for PD-L1 status; data were available for 1010 of those patients. The majority of patients (*n* = 1010/1073, 95%) were tested for PD-L1 status at advanced diagnosis (Table 2). PD-L1 expression ≥ 50% and PD-L1 expression of 1–49% were found in 36.0% and 40.9% of all 1010 patients, and 40.3% and 37.6% of 865 patients receiving 1L at the time of data collection, respectively.

**Table 2:** PD-L1 expression at advanced NSCLC diagnosis for patients currently with *EGFR*-WT/*ALK*-WT mNSCLC

	**Total population**	**1L treatment at data collection**
PD-L1% expression, n (%)		
n	1010	865
< 1	233 (23.1)	191 (22.1)
1 – 49	413 (40.9)	325 (37.6)
≥ 50	364 (36.0)	349 (40.3)

*ALK* Anaplastic lymphoma kinase, *EGFR* Epidermal growth factor receptor, *EGFR-WT/ALK-WT, i.e.* no sensitising EGFR mutation or ALK translocation; wild type, *mNSCLC* metastatic non-small cell lung cancer, *PD-L1* Programmed death ligand 1, *1L* first line

Overall, 1021 patients were tested for PD-L1 status; data were available for 1010 patients

#### Treatment of patients with EGFR-WT/ALK-WT mNSCLC

Among the total *EGFR*-WT/*ALK*-WT mNSCLC population (*n* = 1073), advanced treatment was mostly chemotherapy only (39.2%) followed by immune-oncology monotherapy (IO; 35.0%) (Table 3).

**Table 3:** 1L Treatments ever received for aNSCLC by patients currently with *EGFR*-WT/*ALK*-WT mNSCLC

	**Total population**	**1L treatment at data collection**
1L aNSCLC^a^ Treatment group n (%)		
n	1073	915
IO monotherapy	330 (30.8)	320 (35.0)
IO + chemotherapy	270 (25.2)	258 (28.2)
IO + non-chemotherapy	0 (0.0)	0 (0.0)
Chemotherapy only	421 (39.2)	300 (32.8)
Chemotherapy combination	30 (2.8)	17 (1.9)
Targeted	15 (1.4)	13 (1.4)
Other	7 (0.7)	7 (0.8)

*ALK* Anaplastic lymphoma kinase, *aNSCLC* advanced non-small cell lung cancer, *EGFR* Epidermal growth factor receptor, *EGFR-WT/ALK-WT, i.e.* no sensitising EGFR mutation or ALK translocation; wild type, *IO* Immuno-oncology, *mNSCLC* metastatic non-small cell lung cancer, *1L* first line

^a^aNSCLC treatment lines have been used due the design of the survey focusing on this timepoint

Some patients had aNSCLC treatments before being diagnosed with metastases, although this information is limited and the outcomes for 1L metastatic NSCLC are not relevant

Of 158 patients who had progressed beyond 1L therapy (i.e. 2L +), the mean (SD) time to 1L treatment discontinuation of 5.1 (4.3) months. The full course of 1L treatment was completed as intended by 75.9% of patients, and a complete response was achieved by 6.7% and a partial response by 69.2% of patients. Disease progression was reported for 73.7% of 38 patients who discontinued their 1L treatment early.

#### Demographic and clinical characteristics by 1L treatment group of patients with EGFR-WT/ALK-WT mNSCLC

To stratify patients by treatment, 1L treatments were grouped by class. Among the 1L treatment groups (total population *n* = 1086), 39.6% of patients were receiving chemotherapy only, 30.7% were receiving IO, 25.0% IO + chemotherapy, 2.8% chemotherapy combination (multiple chemotherapy drugs), and 1.4% were receiving targeted therapy at the time of data collection (Table 4). The majority of these patients (≥ 55.6%) had adenocarcinoma. There were few patients in the ‘other’ treatment group (*n* = 7) and this group generally showed different disease characteristics from the other groups.

**Table 4:** Demographic and clinical characteristics of patients with *EGFR*-WT/*ALK*-WT mNSCLC by 1L treatment group

	**Total population**	**1L Treatment group**						***p*** **value**
		**IO monotherapy**	**IO + chemotherapy**	**Chemotherapy only**	**Chemotherapy combination**	**Targeted therapy**	**Other**	
Age, years								
n	1086	333	271	430	30	15	7	< 0.0001
Mean (SD)	66.2 (8.9)	67.5 (8.6)	62.9 (8.3)	67.1 (8.7)	65.1 (7.9)	62.9 (13.2)	82.4 (6.5)	
Sex, n (%)								
n	1086	333	271	430	30	15	7	0.1405
Male	710 (65.4)	221 (66.4)	167 (61.6)	289 (67.2)	21 (57.1)	6 (40.0)	6 (85.7)	
Female	376 (34.6)	112 (33.6)	104 (38.4)	141 (32.8)	9 (30.0)	9 (60.0)	1 (14.3)	
Current disease stage, n (%)								
n	1086	333	271	430	30	15	7	0.7405
Stage IVa	335 (30.8)	96 (28.8)	90 (33.2)	136 (31.6)	8 (26.7)	4 (26.7)	1 (14.3)	
Stage IVb	751 (69.2)	237 (71.2)	181 (66.8)	294 (68.4)	22 (73.3)	11 (73.3)	6 (85.7)	
NSCLC histology, n (%)								
n	1086	333	271	430	30	15	7	< 0.0001
Adenocarcinoma	689 (63.4)	200 (60.1)	215 (79.3)	239 (55.6)	22 (73.3)	11 (73.3)	2 (28.6)	
Squamous cell carcinoma	361 (33.2)	121 (36.3)	50 (18.5)	177 (41.2)	5 (16.7)	3 (20.0)	5 (71.4)	
Large cell carcinoma	24 (2.3)	7 (2.1)	4 (1.5)	9 (2.1)	3 (10.0)	1 (6.7)	0 (0.0)	
Other	12 (1.1)	5 (1.5)	2 (0.7)	5 (1.2)	0 (0.0)	0 (0.0)	0 (0.0)	
Primary site of metastases (> 5%), n (%)								
n	1086	333	271	430	30	15	7	
Contralateral lung	511 (47.1)	164 (49.2)	122 (45.0)	200 (46.5)	17 (56.7)	5 (33.3)	3 (42.9)	0.6323
Lymphatic system/lymph nodes (any)	368 (33.9)	105 (31.5)	87 (32.1)	155 (36.0)	13 (43.3)	5 (33.3)	3 (42.9)	0.6154
Bone	359 (33.1)	111 (33.3)	84 (31.0)	143 (33.3)	14 (46.7)	6 (40.0)	1 (14.3)	0.481
Pleura	259 (23.8)	73 (21.9)	68 (25.1)	110 (25.6)	5 (16.7)	3 (20.0)	0 (0.0)	0.4422
Liver	258 (23.8)	75 (22.5)	67 (24.7)	104 (24.2)	5 (16.7)	6 (40.0)	1 (14.3)	0.5745
Adrenal glands	224 (20.6)	65 (19.5)	63 (23.2)	86 (20.0)	6 (20.0)	2 (13.3)	2 (28.6)	0.8131
Brain	80 (7.4)	20 (6.0)	19 (7.0)	36 (8.4)	2 (6.7)	0 (0.0)	3 (42.9)	0.0077
Current ECOG PS score, n (%)								
n	1083	332	270	429	30	15	7	< 0.0001
0	240 (22.2)	91 (27.4)	79 (29.3)	65 (15.2)	2 (6.7)	3 (20.0)	0 (0.0)	
1	644 (59.5)	205 (61.7)	166 (61.5)	240 (55.9)	21 (70.0)	11 (73.3)	1 (14.3)	
2	150 (13.9)	30 (9.0)	17 (6.3)	99 (23.1)	3 (10.0)	0 (0.0)	1 (14.3)	
3	27 (2.5)	4 (1.2)	3 (1.1)	15 (3.5)	1 (3.3)	1 (6.7)	3 (42.9)	
4	22 (2.0)	2 (0.6)	5 (1.9)	10 (2.3)	3 (10.0)	0 (0.0)	2 (28.6)	
Current symptoms (most frequent 10), n (%)								
n	1086	333	271	430	30	15	7	
Cough	640 (58.9)	189 (56.8)	151 (55.7)	265 (61.6)	19 (63.3)	12 (80.0)	4 (57.1)	0.2968
Fatigue	476 (43.8)	127 (38.1)	123 (45.4)	200 (46.5)	15 (50.0)	6 (40.0)	5 (71.4)	0.1250
Dyspnoea/breathing complications	460 (42.4)	131 (39.3)	114 (42.1)	190 (44.2)	13 (43.3)	7 (46.7)	5 (71.4)	0.4948
Weight loss	305 (28.1)	75 (22.5)	73 (26.9)	141 (32.8)	9 (30.0)	5 (33.3)	2 (28.6)	0.0684
Loss of appetite	289 (26.6)	65 (19.5)	77 (28.4)	132 (30.7)	9 (30.0)	3 (20.0)	3 (42.9)	0.0146
Bone pain	264 (24.3)	68 (20.4)	66 (24.4)	112 (26.0)	12 (40.0)	3 (20.0)	3 (42.9)	0.1124
Chest pain	277 (25.5)	63 (18.9)	66 (24.4)	127 (29.5)	13 (43.3)	5 (33.3)	3 (42.9)	0.0029
Persistent cough	223 (20.5)	62 (18.6)	44 (16.2)	106 (24.7)	6 (20.0)	3 (20.0)	2 (28.6)	0.1276
Low mood	175 (16.1)	36 (10.8)	47 (17.3)	85 (19.8)	5 (16.7)	2 (13.3)	0 (0.0)	0.0242
Weak limbs	155 (14.3)	30 (9.0)	34 (12.5)	82 (19.1)	7 (23.3)	1 (6.7)	1 (14.3)	0.0019
Current comorbidities (> 5%), n (%)								
n	1086	333	271	430	30	15	7	
Hypertension	414 (38.1)	125 (37.5)	81 (29.9)	181 (42.1)	18 (60.0)	6 (40.0)	3 (42.9)	0.0047
Chronic pulmonary disease	264 (24.3)	67 (20.1)	57 (21.0)	124 (28.8)	10 (33.3)	2 (13.3)	4 (57.1)	0.007
Dyslipidaemia	169 (15.6)	49 (14.7)	34 (12.5)	79 (18.4)	6 (20.0)	0 (0.0)	1 (14.3)	0.1638
Diabetes without chronic complications	129 (11.9)	50 (15.0)	14 (5.2)	57 (13.3)	5 (16.7)	1 (6.7)	2 (28.6)	0.0024
Peripheral vascular disease	55 (5.1)	17 (5.1)	6 (2.2)	31 (7.2)	0 (0.0)	1 (6.7)	0 (0.0)	0.0566
Current Charlson comorbidity index								
n	1086	333	271	430	30	15	7	< 0.0001
Mean (SD)	0.5 (0.9)	0.4 (0.8)	0.4 (0.8)	0.6 (1.0)	0.8 (1.2)	0.2 (0.4)	1.9 (1.5)	

*ALK* Anaplastic lymphoma kinase, *aNSCLC* advanced non-small cell lung cancer, *ECOG PS* Eastern Cooperative Oncology Group performance status, *EGFR* Epidermal growth factor receptor, *EGFR-WT/ALK-WT, i.e.* no sensitising EGFR mutation or ALK translocation; wild type, *IO* Immuno-oncology, *mNSCLC* metastatic non-small cell lung cancer, *SD* Standard deviation, *1L* First line

Current: time of consultation

ECOG performance status scale: 0, Fully active, able to carry on all pre-disease performance without restriction; 1, Restricted in physically strenuous activity but ambulatory and able to carry out work of a light or sedentary nature, e.g., light house work, office work; 2, Ambulatory and capable of all selfcare but unable to carry out any work activities; up and about more than 50% of waking hours; 3, Capable of only limited selfcare; confined to bed or chair more than 50% of waking hours; 4, Completely disabled; cannot carry on any selfcare; totally confined to bed or chair; 5, Dead

Charlson comorbidity index: index ranges from 0, low risk of mortality attributable to comorbid disease, to ≥ 5, with stepwise increases in the cumulative mortality attributable to comorbid disease

No differences were observed in age, gender and disease stage according to 1L treatment, compared with the total population of *EGFR*-WT/*ALK*-WT mNSCLC patients (Table 4). There was a significant difference between all treatment groups in the proportions of patients with brain metastases (p = 0.0077). ECOG PS significantly differed between all treatment groups (*p* < 0.0001); the majority of patients in all treatment groups (chemotherapy only, IO, IO + chemotherapy, chemotherapy combination, and targeted therapy) had a PS of 1,excluding the ‘other’ treatment group (PS ≥ 3 in three of seven patients). The proportions of patients with each common symptom were similar among all treatment groups, with the exception of loss of appetite (p = 0.0146), chest pain (p = 0.0029) and weak limbs (p = 0.0019). There was a difference between groups in comorbid hypertension (p = 0.0047), chronic pulmonary disease (p = 0.007), and diabetes without chronic complications (p = 0.0024); current Charlson comorbidity index was ≤ 0.8 for most treatment groups and 1.9 for the ‘other’ patient group (*p* < 0.0001 all treatment groups).

#### 1L Treatment by histology of patients with EGFR-WT/ALK-WT mNSCLC

There were differences in the 1L treatment received (*p* < 0.0001) by patients according to the histology of their NSCLC (squamous cell carcinoma, adenocarcinoma, large cell carcinoma and ‘other’) (Table 5); chemotherapy only was most commonly received by patients with adenocarcinoma, squamous cell carcinoma and large cell carcinoma. The next most common treatment was IO for patients with squamous cell carcinoma and large cell carcinoma, and IO + chemotherapy for patients with adenocarcinoma. Treatment response and reason for discontinuation were similar between patient histology groups. The majority of patients who completed 1L treatment achieved a partial response and the most common reason for discontinued treatment was disease progression.

**Table 5:** 1L Treatment of patients with *EGFR*-WT/*ALK*-WT mNSCLC by histology

	**Total population**	**Histology**				***p* value**
		**Squamous cell carcinoma**	**Adenocarcinoma**	**Large-cell carcinoma**	**Other**	
1L mNSCLC^a^ Treatment group, n (%)						
n	1086	361	689	24	12	
IO monotherapy	333 (30.7)	121 (33.5)	200 (29.0)	7 (29.2)	5 (41.7)	< 0.0001
IO + chemotherapy	271 (25.0)	50 (13.9)	215 (31.2)	4 (16.7)	2 (16.7)	
IO + non-chemotherapy	0 (0.0)	0 (0.0)	0 (0.0)	0 (0.0)	0 (0.0)	
Chemotherapy only	430 (39.6)	177 (49.0)	239 (34.7)	9 (37.5)	5 (41.7)	
Chemotherapy combination	30 (2.8)	5 (1.4)	22 (3.2)	3 (12.5)	0 (0.0)	
Targeted	15 (1.4)	3 (0.8)	11 (1.6)	1 (4.2)	0 (0.0)	
Other	7 (0.6)	5 (1.4)	2 (0.3)	0 (0.0)	0 (0.0)	
1L Full course completed^b^, n (%)						
n	190	54	127	6	3	
Yes	149 (78.4)	45 (83.3)	96 (75.6)	5 (83.3)	3 (100.0)	
1L Response achieved^b^, n (%)						
n	149	45	96	5	3	
Full response	14 (9.4)	2 (4.4)	10 (10.4)	1 (20.0)	1 (33.3)	0.2202
Partial response	106 (71.1)	30 (66.7)	72 (75.0)	3 (60.0)	1 (33.3)	
No response	29 (19.5)	13 (28.9)	14 (14.6)	1 (20.0)	1 (33.3)	
Reasons for 1L treatment early discontinuation^b^, n (%)						
n	41	9	31	1	0	
Not responding	3 (7.3)	1 (11.1)	2 (6.5)	0 (0.0)	0 (0.0)	0.8589
Disease progression	28 (68.3)	5 (55.6)	22 (71.0)	1 (100.0)	0 (0.0)	0.5377
Side-effects	8 (19.5)	3 (33.3)	5 (16.1)	0 (0.0)	0 (0.0)	0.4577
Other	4 (9.8)	2 (22.2)	2 (6.5)	0 (0.0)	0 (0.0)	0.3532
Time to 1L treatment discontinuation, months^b^						
n	185	51	126	5	3	0.8233
Median (range)	4 (0.7, 33.7)	3.6 (1.3, 17.4)	4 (0.7, 33.7)	3 (2.0, 13.0)	4 (2.1, 6.0)	

*ALK* Anaplastic lymphoma kinase, *aNSCLC* advanced non-small cell lung cancer, *EGFR* Epidermal growth factor receptor, *EGFR-WT/ALK-WT, i.e.* no sensitising EGFR mutation or ALK translocation; wild type, *IO* Immuno-oncology, *mNSCLC* metastatic non-small cell lung cancer, *SD* Standard deviation, *1L* first line, *2L* + second line treatment and beyond

^a^mNSCLC treatment lines have been used due the design of the survey focusing on this timepoint

^b^Data from patients who had progressed beyond 1L and are currently 2L +

#### Disease characteristics and 1L treatment of patients with EGFR-WT/ALK-WT mNSCLC by PD-L1 status

In patients with *EGFR*-WT/*ALK*-WT mNSCLC (*n* = 1021), prevalence of PD-L1 < 1%, 1–49% and ≥ 50% expression was 22.9%, 41.1%, and 35.9% patients, respectively (Table 6).

**Table 6:** Disease characteristics and treatment of patients with *EGFR*-WT/*ALK*-WT mNSCLC by PD-L1 expression

	**Total population**	**PD-L1% expression**			***p* value**
		** < 1**	**1 – 49**	** ≥ 50**	
*Disease Stage and Histology*					
Current disease stage, n (%)					0.7423
n	1021	234	420	367	
Stage IVA	307 (30.1)	73 (31.2)	129 (30.7)	105 (28.6)	
Stage IVB	714 (69.9)	161 (68.8)	291 (69.3)	262 (71.4)	
NSCLC histology, n (%)					0.9400
n	1021	234	420	367	
Squamous cell carcinoma	333 (32.6)	79 (33.8)	131 (31.2)	123 (33.5)	
Adenocarcinoma	657 (64.3)	148 (63.2)	277 (66.0)	232 (63.2)	
Large cell carcinoma	20 (2.0)	4 (1.7)	9 (2.1)	7 (1.9)	
Other	11 (1.1)	3 (1.3)	3 (0.7)	5 (1.4)	
*1L mNSCLC*^a^* Treatment group*					
1L treatment group, n (%)					
n	1021	234	420	367	
IO monotherapy	330 (32.3)	6 (2.6)	27 (6.4)	297 (80.9)	< 0.0001
IO + chemotherapy	268 (26.2)	62 (26.5)	155 (36.9)	51 (13.9)	
IO + non-chemotherapy	0 (0.0)	0 (0.0)	0 (0.0)	0 (0.0)	
Chemotherapy only	385 (37.7)	146 (62.4)	222 (52.9)	17 (4.6)	
Chemotherapy combination	25 (2.4)	16 (6.8)	8 (1.9)	1 (0.3)	
Targeted	11 (1.1)	3 (1.3)	7 (1.7)	1 (0.3)	
Other	2 (0.2)	1 (0.4)	1 (0.2)	0 (0.0)	
1L Full course completed, n (%)					
n	174	51	105	18	
Yes	134 (77.0)	41 (80.4)	81 (77.1)	12 (66.7)	
1L Response achieved, n (%)					0.0828
n	134	41	81	12	
Full response	11 (8.2)	5 (12.2)	4 (4.9)	2 (16.7)	
Partial response	95 (70.9)	23 (56.1)	64 (79.0)	8 (66.7)	
No response	28 (20.9)	13 (31.7)	13 (16.0)	2 (16.7)	
Reasons for 1L treatment early discontinuation, n (%)					
n	40	10	24	6	
Not responding	3 (7.5)	1 (10.0)	1 (4.2)	1 (16.7)	0.5485
Disease progression	28 (70.0)	5 (50.0)	19 (79.2)	4 (66.7)	0.2349
Side-effects	7 (17.5)	3 (30.0)	3 (12.5)	1 (16.7)	0.4722
Other	4 (10.0)	1 (10.0)	2 (8.3)	1 (16.7)	0.831
Time to 1L treatment discontinuation, months^b^					0.1122
n	169	51	100	18	
Median (Range)	4.0 (0.7, 33.7)	3.9 (2.0, 17.4)	4.0 (0.7, 33.7)	4.5 (1.4, 25.0)	

*ALK* Anaplastic lymphoma kinase, *aNSCLC* advanced non-small cell lung cancer, *EGFR* Epidermal growth factor receptor, *EGFR-WT/ALK-WT, i.e.* no sensitising EGFR mutation or ALK translocation; wild type, *IO* Immuno-oncology, *mNSCLC* Metastatic non-small cell lung cancer, *NSCLC* Non-small cell lung cancer, *PD-L1* Programmed death ligand 1, *SD* Standard deviation, *1L* First line, *2L* + second line treatment and beyond

^a^mNSCLC treatment lines have been used due the design of the survey focusing on this timepoint

^b^Data from patients who had progressed beyond 1L and are currently 2L +

In the 1L treatment setting, the majority of patients with PD-L1 expression of < 1% (62.4% of 234 patients) and 1–49% (52.9% of 420 patients) received chemotherapy only; the majority of patients with PD-L1 ≥ 50% received IO (80.9% of 367 patients).

The median time to 1L treatment discontinuation in the 1L PD-L1-tested *EGFR*-WT/*ALK*-WT mNSCLC population who had progressed beyond 1L treatment was 4.0 months (3.9, 4.0, and 4.5 months for patients with PD-L1 expression of < 1%, 1–49%, and ≥ 50%, respectively). The full course of 1L treatment was completed as intended by 77.0% patients (80.4%, 77.1% and 66% of patients with PD-L1 expression of < 1%, 1–49%, and ≥ 50%, respectively), a complete response was achieved by 8.2% of patients and a partial response by 70.9% of patients. No response was reported for twice as many patients with PD-L1 expression of < 1% (31.7%) versus 1–49% (16.0%) and ≥ 50% (16.7%). For the overall 1L *EGFR*-WT/*ALK*-WT mNSCLC population for who reasons for early 1L treatment discontinuation were reported (*n* = 40), disease progression was given as a reason in 70.0% of patients, with no statistical difference between the PD-L1 expression groups.

#### Quality of life of patients with EGFR-WT/ALK-WT mNSCLC receiving 1L

Patient-reported EQ-5D VAS, EQ-5D utility index, and FACT are reported in Table 7. For the overall population of 260 patients with *EGFR*-WT/*ALK*-WT mNSCLC who completed a patient-reported questionnaire, patient-reported EQ-5D VAS mean (SD) score was 67.3 (16.5), which was lower than the mean normative reference value for France (76.8) [25]. Mean (SD) VAS scores ranged from 60.7 (19.86) for France to 71.2 (16.94) for Spain.

**Table 7:** Quality of life in patients with *EGFR*-WT/*ALK*-WT mNSCLC in the 1L setting

	**Total population**	**France**	**Germany**	**Italy**	**Spain**	**UK**
EQ-5D VAS						
n	260	40	57	46	82	35
Mean (SD)	67.3 (16.5)	60.7 (19.9)	66.5 (15.2)	64.5 (13.1)	71.2 (16.9)	70.5 (15.0)
EQ-ED utility index score (French 5L)						
n	256	41	54	46	80	35
Mean (SD)	0.9 (0. 2)	0.8 (0.3)	0.9 (0.1)	0.9 (0.2)	0.9 (0.1)	0.9 (0.1)
FACT-G score (range 0–108)						
n	253	40	51	46	81	35
Mean (SD)	62.8 (15.5)	57.4 (17.4)	65.1 (15.4)	60.1 (10.7)	64.8 (17.7)	64.8 (11.5)
FACT-L score (range 0–136)						
n	253	40	51	46	81	35
Mean (SD)	80 (18.8)	72.7 (21.4)	83.0 (18.2)	76.9 (13.3)	82.5 (21.3)	82.4 (14.1)
FACT-Lung Cancer Subscale score (range 0–28)						
n	256	40	53	46	82	35
Mean (SD)	17.2 (4.4)	15.3 (5.0)	17.9 (4.4)	16.8 (3.5)	17.6 (4.5)	17.6 (3.7)
FACT-Trial Outcome Index score (range 0–84)						
n	254	40	52	46	81	35
Mean (SD)	48.9 (12.9)	43.0 (15.7)	50.0 (11.7)	47.4 (9.1)	51.2 (13.9)	50.4 (11.3)
FACT-Physical Well Being score (range 0–28)						
n	258	40	56	46	81	35
Mean (SD)	19.5 (5.2)	17.1 (7.0)	20.1 (5.2)	20.4 (3.6)	20.2 (4.9)	18.7 (4.5)
FACT-Social/family Well Being score (range 0–28)						
n	255	40	53	46	81	35
Mean (SD)	17.4 (5.8)	17.0 (6.0)	18.8 (6.7)	14.9 (4.5)	17.9 (6.0)	18.3 (4.1)
FACT-Emotional Well Being score (range 0–24)						
n	254	40	52	46	81	35
Mean (SD)	13.7 (4.4)	12.6 (4.5)	14.4 (4.8)	14.6 (3.7)	13.4 (4.6)	13.7 (3.6)
FACT-Functional Well Being score (range 0–28)						
n	255	40	53	46	81	35
Mean (SD)	12.1 (5.8)	10.7 (5.9)	11.8 (5.6)	10.3 (4.1)	13.3 (6.5)	14.1 (5.1)

*ALK* Anaplastic lymphoma kinase, *EGFR* Epidermal growth factor receptor, *EGFR-WT/ALK-WT, i.e.* no sensitising EGFR mutation or ALK translocation; wild type; *FACT* Functional Assessment of Cancer Therapy, *FACT-G* FACT-General, *FACT-L* FACT-Lung, *mNSCLC* metastatic non-small cell lung cancer, *SD* Standard deviation, *VAS* Visual analogue scale, *1L* first line

Patient mean (SD) EQ-5D-5L utility score was 0.86 (0.17), which was in line with the mean normative reference value for France (0.87) [25]. Mean EQ-5D-5L utility scores ranged from 0.77 (0.29) for France to 0.90 (0.12) for UK. The MCID between patients in France and patients in Germany, Italy, Spain and the UK for EQ-5D utility index was > 0.082, and was > 0.7 between patients in all evaluated countries for EQ-5D VAS.

Patient mean (SD) FACT-G score was 62.8 (15.5), which was noticeably lower than the reported mean US population normative reference value of 80.1 [32]. Mean (SD) FACT-G scores ranged from 57.4 (17.40) for France to 65.1 (15.41) for Germany. Patient mean (SD) FACT-L score was 80.0 (18.8). Mean FACT-L scores ranged from 72.7 (21.4) for France to 83.0 (18.2) for Germany. The MCID was > 2 points for FACT-Lung Cancer Subscale score between patients in France and patients in Germany, Spain and the UK, but not between patients in Germany, Italy, Spain and the UK.

### Oversample

#### Physician and patient populations

For the retrospective oversample, 252 oncologists/pulmonologists (France, *n* = 51; Germany, *n* = 50; Italy, *n* = 50; Spain, *n* = 50, UK, *n* = 51) completed retrospective patient record forms for 2537 patients with *EGFR*-WT mNSCLC (France: *n* = 504, [19.9%], Germany: *n* = 501 [19.7%], Italy: *n* = 501 [19.7%], Spain: *n* = 515 [20.3%], UK: *n* = 516 [20.3%]. Of these, 2373 patients were also *ALK*-WT (*EGFR*-WT/*ALK*-WT, France: *n* = 479 [20.2%], Germany: *n* = 479 [20.2%], Italy: *n* = 460 [19.4%], Spain: *n* = 491 [20.7%], UK: *n* = 464, [19.6%]).

The retrospective oversample analysis was based around the emergence of COVID-19 in Europe and examined effects of the virus and ‘lockdown’ on the treatment and management of mNSCLC. The pre-COVID-19 period was defined as patients diagnosed from 1^st^ September 2019 to 29^th^ February 2020, and the period during COVID-19 was defined as patients diagnosed from 1^st^ March 2020 (a date where all five European countries were in lockdown due to COVID-19) up to when data collection ended (August 2021). The total sample for analysis included 2373 *EGFR*-WT/*ALK*-WT patients; 1148 patients diagnosed in the pre-COVID-19 population and 1225 patients diagnosed in the population sampled during COVID-19 (Fig. 1).

#### Demographics and clinical characteristics of patients with EGFR-WT/ALK-WT mNSCLC

Patient characteristics for the *EGFR*-WT/*ALK*-WT mNSCLC population split by the period in which patients were diagnosed (pre-COVID-19 and during COVID-19) are shown in Table 8.

**Table 8:** Demographic and clinical characteristics of patients with *EGFR*-WT/*ALK*-WT mNSCLC, diagnosed pre- and during the COVID-19 pandemic

	**Total population**	**Diagnostic period**	
		**Pre-COVID-19**	**During COVID-19**
Age, years			
n	2370	1147	1223
Mean (SD)	66.4 (8.9)	66.4 (8.7)	66.4 (9.0)
Sex, n (%)			
n	2373	1148	1225
Male	1584 (66.8)	776 (67.6)	808 (66.0)
Female	789 (33.2)	372 (32.4)	417 (34.0)
Disease stage at mNSCLC diagnosis, n (%)			
n	2373	1148	1225
Stage IVa	935 (39.4)	478 (41.6)	475 (37.3)
Stage IVb	1438 (60.6)	670 (58.4)	768 (62.7)
NSCLC histology, n (%)			
n	2373	1148	1225
Adenocarcinoma	1513 (63.8)	730 (63.6)	783 (63.9)
Squamous cell carcinoma	786 (33.1)	386 (33.6)	400 (32.7)
Large cell carcinoma	54 (2.3)	23 (2.0)	31 (2.5)
Other	17 (0.7)	7 (0.6)	10 (0.8)
Don’t know/not assessed	3 (0.1)	2 (0.2)	1 (0.1)
Comorbidities at data collection (≥ 5%), n (%)			
n	2373	1148	1225
Hypertension	992 (41.8)	478 (41.6)	514 (42.0)
Dyslipidaemia	504 (21.2)	246 (21.4)	258 (21.1)
Chronic pulmonary disease	485 (20.4)	249 (21.7)	236 (19.3)
Diabetes without chronic complications	345 (14.5)	176 (15.3)	169 (13.8)
Peripheral vascular disease	165 (7.0)	80 (7.0)	85 (6.9)
Mild liver disease	119 (5.0)	55 (4.8)	64 (5.2)
Tests/assessments in mNSCLC diagnosis (> 5%), n (%)			
n	2370	1148	1225
Biopsy	2090 (88.1)	1004 (87.5)	1086 (88.7)
Blood tests	2058 (86.7)	1001 (87.2)	1057 (86.3)
CT scan of chest	1955 (82.4)	961 (83.7)	994 (81.1)
Bronchoscopy	1541 (64.9)	753 (65.5)	788 (64.3)
FDG PET scan	1447 (61.0)	705 (61.4)	742 (60.6)
X-ray	1110 (46.8)	531 (46.3)	579 (47.3)
Pulmonary function tests	1032 (43.5)	480 (41.8)	552 (45.1)
MRI	812 (34.2)	383 (33.4)	429 (35.0)
Radioisotope/bone scan	805 (33.9)	399 (34.8)	406 (33.1)
Ultrasound	479 (20.2)	236 (20.6)	243 (19.8)
PD-L1 expression level			
n	2239	1075	1164
< 1%	440 (19.7)	222 (20.7)	218 (18.7)
1%-49%	1038 (46.4)	505 (47.0)	533 (45.8)
≥ 50%	761 (34)	348 (32.4)	413 (35.5)
PD-L1 result obtained prior to mNSCLC treatment initiation, n (%)			
n	2252	1081	1171
Yes	2141(95.1)	1027 (95.0)	1114 (95.1)
No	101 (4.5)	48 (4.4)	53 (4.5)
Don’t know	10 (0.4)	6 (0.6)	4 (0.3)

*ALK* Anaplastic lymphoma kinase, *CT* Computerized tomography, *EGFR* Epidermal growth factor receptor, *EGFR-WT/ALK-WT, i.e.* no sensitising EGFR mutation or ALK translocation; wild type, *FDG PET* Fluorodeoxyglucose*-*positron emission tomography*, mNSCLC* metastatic non-small cell lung cancer, *MRI* Magnetic resonance imaging, *NSCLC* Non-small cell lung cancer, *PD-L1* Programmed death ligand 1, *SD* Standard deviation

At the time of most recent consultation, patients’ mean age was 66.4 (8.9) years and 1584 (66.8%) were male. At the time of mNSCLC diagnosis, 935 (39.4%) had stage IVa disease and 1438 (60.6%) had stage IVb disease. Adenocarcinoma (*n* = 1513; 63.8%) and squamous cell carcinoma (*n* = 786; 33.1%) were the most prevalent NSCLC histological types. The most common comorbid conditions at time of data collection across the COVID cohorts were hypertension (41.8%), dyslipidaemia (21.1%), and chronic pulmonary disease (20.4%). Characteristics of patients diagnosed pre-COVID and during COVID-19 seemed to be similar.

The majority of patients had undergone a biopsy (*n* = 2090; 88.1%), blood tests (*n* = 2058; 86.7%), a CT chest scan (*n* = 1955; 82.4%), and bronchoscopy (*n* = 1541; 64.9%) during mNSCLC diagnosis in both diagnosis periods. PD-L1 status at mNSCLC diagnosis was established in 2239 (94.9%) of 2373 patients, and of these 2239 patients who had their PD-L1 expression level determined, 19.7% had an expression < 1%, 46.4% had an expression level of 1%-49% and 34% had an expression level of ≥ 50%. The PD-L1 test result was obtained prior to treatment initiation in 2141 (95.1%) patients. There seemed to be no notable differences in PD-L1 parameters between the pre- and during COVID cohorts.

#### COVID-19 status of patients with EGFR-WT/ALK-WT mNSCLC

A total of 1268 patients with *EGFR*-WT/*ALK*-WT mNSCLC had at least one COVID-19 test; these patients had undergone a mean (SD) of 5.8 (9.1) COVID-19 tests, ranging from 2.7 (2.9) in Spain to 11.8 (17.3) in Germany (Table 9). The majority of patients had tested negative on their most recent COVID test (*n* = 1231; 95.7%), ranging from 90.0% (*n* = 261) in Spain to 99.6% (*n* = 268) in Germany. Over half (*n* = 727; 56.5%) of patients had taken their most recent test more than two weeks prior to the consultation, and 241 (18.7%) patients had tested within the last two weeks.

**Table 9:** COVID-19 status of patients with *EGFR*-WT/*ALK*-WT mNSCLC

	**Total population**	**France**	**Germany**	**Italy**	**Spain**	**UK**
Base, n	2373	479	479	460	491	464
Patients who had at least one COVID-19 test, n (%)	1286 (54.2)	227 (47.4)	269 (56.2)	276 (60.0)	290 (59.1)	224 (48.3)
COVID-19 tests per patient						
n	1286	227	269	276	290	224
Mean (SD)	5.8 (9.1)	3.3 (3.0)	11.8 (17.3)	5.8 (4.3)	2.7 (2.9)	5.4 (4.3)
Most recent COVID-19 test result, n (%)						
n	1286	227	269	276	290	224
Positive	55 (4.3)	13 (5.7)	1 (0.4)	8 (2.9)	29 (10.0)	4 (1.8)
Negative	1231 (95.7)	214 (94.3)	268 (99.6)	268 (97.1)	261 (90.0)	220 (98.2)
Time of most recent COVID-19 test to data collection, n (%)						
n	1286	227	269	276	290	224
Within the last week	176 (13.7)	10 (4.4)	80 (29.7)	42 (15.2)	16 (5.5)	28 (12.5)
Within the last two weeks	241 (18.7)	23 (10.1)	58 (21.6)	73 (26.4)	28 (9.7)	59 (26.3)
More than two weeks ago	727 (56.5)	175 (77.1	83 (30.9	132 (47.8	227 (78.3)	110 (49.1
Don’t know	142 (11.0)	19 (8.4)	48 (17.8)	29 (10.5)	19 (6.6)	27 (12.1)
COVID-19 status at data collection, n (%)						
n	2373	479	479	460	491	464
Confirmed positive diagnosis	40 (1.7)	3 (0.6)	5 (1.0)	4 (0.9)	14 (2.9)	14 (3.0)
Currently considered negative	1787 (75.3)	354 (73.9)	384 (80.2)	383 (83.3)	354 (72.1)	312 (67.2
Don’t know	404 (17.0)	99 (20.7)	79 (16.5)	41 (8.9)	77 (15.7)	108 (23.3)

*ALK* Anaplastic lymphoma kinase, *EGFR* Epidermal growth factor receptor, *EGFR-WT/ALK-WT, i.e.* no sensitising EGFR mutation or ALK translocation; wild type, *mNSCLC* metastatic non-small cell lung cancer, *SD* Standard deviation

At data collection, 1787 (75.3%) of patients were considered to be negative for COVID-19. The last COVID-19 test could have been any time up to the day of data collection, and so the lower percentage of tests considered to be negative was due to more patients having an unknown COVID-19 status at the point of consultation. A total of 40 (1.7%) patients had a current confirmed case of COVID-19 at time of data collection.

#### Impact of COVID-19 on treatment and management* mNSCLC*

Physicians reported that the management of 34.7% (*n* = 823) of patients had been impacted as a result of the COVID-19 pandemic (Table 10). The impact on management affected 78.7% (*n* = 365) of patients in the UK, 32.4% (*n* = 159) in Spain, 22.8% (*n* = 109) in France, 20.9% (*n* = 96) in Italy, and 19.6% (*n* = 94) in Germany.

**Table 10:** Impact of COVID-19 on treatment and management of *EGFR*-WT/*ALK*-WT mNSCLC

	**Total population**	**France**	**Germany**	**Italy**	**Spain**	**UK**
General effects of COVID-19 on patient management, n (%)						
n	2373	479	479	460	491	464
No impact on management	1550 (65.3)	370 (77.2)	385 (80.4)	364 (79.1)	332 (67.6)	99 (21.3)
Moving to video/telephone consultation	474 (20.0)	69 (14.4)	2 (0.4)	22 (4.8)	87 (17.7)	294 (63.4)
Reduced consultation frequency	354 (14.9)	39 (8.1)	58 (12.1)	61 (13.3)	93 (18.9)	103 (22.2)
Fewer tests/investigations	133 (5.6)	15 (3.1)	11 (2.3)	24 (5.2)	39 (7.9)	44 (9.5)
Patient missed arranged consultations	129 (5.4)	24 (5.0)	17 (3.5)	15 (3.3)	47 (9.6)	26 (5.6)
Reduced treatment monitoring	67 (2.8)	0 (0.0)	11 (2.3)	14 (3)	23 (4.7)	19 (4.1)
Impacted choice of therapy and/or frequency	23 (1.0)	4 (0.8)	2 (0.4)	1 (0.2)	4 (0.8)	12 (2.6)
Delayed/cancelled surgery	2 (0.1)	1 (0.2)	0 (0.0)	0 (0.0)	1 (0.2)	0 (0.0)
Other	17 (0.7)	2 (0.4)	9 (1.9)	2 (0.4)	4 (0.8)	0 (0.0)
Type of current consultation, n (%)						
n	2373	479	479	460	491	464
Face-to-face	2080 (87.7)	466 (97.3)	460 (96.0)	443 (96.3)	455 (92.7)	256 (55.2)
Telephone	200 (8.4)	5 (1.0)	11 (2.3)	4 (0.9)	24 (4.9)	156 (33.6)
Video/online	80 (3.4)	8 (1.7)	8 (1.7)	9 (2.0)	4 (0.8)	51 (11.0)
Other	13 (0.5)	0 (0.0)	0 (0.0)	4 (0.9)	8 (1.6)	1 (0.2)
Prescribed treatment change due to COVID-19, n (%)						
n	2373	479	479	460	491	464
Yes	85 (3.6)	12 (2.5)	2 (0.4)	5 (1.1)	22 (4.5)	44 (9.5)
No	2288 (96.4)	467 (97.5)	477 (99.6)	455 (98.9)	469 (95.5)	420 (90.5)

*ALK* Anaplastic lymphoma kinase, *EGFR* Epidermal growth factor receptor, *EGFR-WT/ALK-WT, i.e.* no sensitising EGFR mutation or ALK translocation; wild type, *mNSCLC* metastatic non-small cell lung cancer

For the total population of patients with *EGFR*-WT/*ALK*-WT mNSCLC (*n* = 2373), a reduction in frequency of consultation was reported for 14.9% (range: 8.1% in France to 22.2% in the UK). Additionally, there was a move to video/telephone consultations for 20.0% of patients, which varied widely between countries; from 0.4% of patients in Germany to 63.4% of patients in the UK.

For their most recent consultation, the majority of patients (87.7%) were seen face-to-face with their physician, ranging from 92.7% of patients in Spain, 96% in Germany, 96.3% in Italy, and 97.3% of patients in France. However, the face-to-face consultation rate was 55.2% for patients in the UK, where 33.6% of patients had telephone consultations and 11.0% had consultations by video/online links. In France, Germany, Italy, and Spain, telephone and video/online consultations were held with < 5% and ≤ 2% of patients, respectively.

#### Treatment of patients with EGFR-WT/ALK-WT mNSCLC diagnosed pre- and during COVID-19

Of 2372 patients with *EGFR*-WT/*ALK*-WT mNSCLC, pembrolizumab (53.5%) and carboplatin (45.4%) were the most frequent 1L therapies (either as monotherapy or in combination) used both pre-COVID-19 (*n* = 1147) and during COVID-19 (*n* = 1225). Between the patients diagnosed pre- and during COVID-19, there was little changes in use of the majority of mNSCLC therapies. 1L immunotherapy, either as monotherapy or combination therapy, was prescribed in 64.2% of the population diagnosed during COVID-19 and 47.8% of patients diagnosed pre-COVID-19; the between-group difference was mostly observed in immunotherapy combination therapy (Table 11).

**Table 11:** Treatment of patients with *EGFR*-WT/*ALK*-WT mNSCLC diagnosed pre- and during the COVID-19 pandemic

	**Total population**	**Diagnostic period**	
		**Pre-COVID-19**	**During COVID-19**
Treatment line at data collection, n (%)			
n	2362	1143	1219
First line	1350 (57.2)	498 (43.6)	852 (69.9)
Second line	847 (35.9)	508 (44.4)	339 (27.8)
Third line	165 (7.0)	137 (12.0)	28 (2.3)
1L Treatment group n (%)			
n	2372	1147	1225
IO monotherapy	672 (28.3)	303 (26.4)	369 (30.1)
IO + chemotherapy	662 (27.9)	245 (21.4)	417 (34.0)
IO + non-chemotherapy	1 (0.0)	1 (0.1)	0 (0.0)
Chemotherapy only	852 (35.9)	516 (45.0)	336 (27.4)
Chemotherapy combination	132 (5.6)	66 (5.8)	66 (5.4)
Targeted therapy	37 (1.6)	10 (0.9)	27 (2.2)
Other	16 (0.7)	6 (0.5)	10 (0.8)
1L Treatment use (most frequent 10), n (%)			
n	2372	1147	1225
Pembrolizumab	1270 (53.5)	524 (45.7)	746 (60.9)
Carboplatin	1076 (45.4)	521 (45.4)	555 (45.3)
Pemetrexed	863 (36.4)	442 (38.5)	421 (34.4)
Cisplatin	472 (19.9)	261 (22.8)	211 (17.2)
Paclitaxel	333 (14.0)	150 (13.1)	183 (14.9)
Gemcitabine	224 (9.4)	129 (11.2)	95 (7.8)
Vinorelbine	114 (4.8)	64 (5.6)	50 (4.1)
Bevacizumab	107 (4.5)	53 (4.6)	54 (4.4)
Nab-paclitaxel	65 (2.7)	24 (2.1)	41 (3.3)
Docetaxel	57 (2.4)	29 (2.5)	28 (2.3)

*ALK* Anaplastic lymphoma kinase, *EGFR* Epidermal growth factor receptor, *EGFR-WT/ALK-WT, i.e.* no sensitising EGFR mutation or ALK translocation; wild type, *IO* Immuno-oncology, *mNSCLC* metastatic non-small cell lung cancer, *1L* first line

Specifically, treatment use of pembrolizumab-based treatment was 60.9% of patients diagnosed during COVID-19 and 45.7% of patients diagnosed pre-COVID-19. Conversely, cisplatin was used by 22.8% and 17.2% of patients diagnosed pre-COVID-19 and during COVID-19, respectively, and pemetrexed was used by 38.5% and 34.4% of patients, respectively.

## Discussion

This analysis of real-word patient data evaluated the characteristics and the current diagnostic landscape of patients with *EGFR*/*ALK* mNSCLC across five European countries, and the impact of COVID-19 on the treatment and management of this population. Immunotherapy is considered the standard approach for most patients with newly diagnosed *EGFR*-WT/*ALK*-WT mNSCLC and tumour PD-L1 ≥ 50% [11]. However, although there was indication that use of chemotherapy was being replaced by immunotherapies, chemotherapy-based regimens were frequently prescribed as 1L treatment.

With the evolving 1L treatment landscape and the introduction of immunotherapy, we found approximately similar usage rates of chemotherapy only and IO only, with one quarter of patients treated with IO + chemotherapy, in the current 1L setting.

PD-1 inhibitors play an important role in the treatment of patients with mNSCLC and, alongside their development, predictive biomarker testing for tumour genomic aberrations in such genes as *EGFR* or *ALK*, and PD-L1 expression have become mandatory in most European countries [11]. The likelihood of clinical benefit from anti-PD-1/PD-L1 agents in the 1L and 2L setting is related to the extent of PD-L1 expression on tumour cells [34]. The mandatory treatment threshold of PD-L1 expression for pembrolizumab is ≥ 50% in 1L and ≥ 1% in second line [11]. This analysis demonstrated that over one-third of patients with *EGFR*-WT/*ALK*-WT mNSCLC had PD-L1 expression of ≥ 50%. The majority of biomarker results of patients were received before initiation of 1L treatment and therefore it may be assumed that these results were available to inform the 1L treatment prescription. PD-L1 ≥ 50% is a reimbursement criterium in prescribing IO (pembrolizumab) for a number of markets [35], and therefore would play a significant role in informing 1L treatment.

Anti-PD-1/PD-L1 treatments are considered to be the cornerstone of 1L therapy for patients with aNSCLC lacking a targetable driver alteration, prescribed as monotherapy for patients with aNSCLC with tumour cell PD-L1 expression ≥ 50%, and typically as combination regimens with platinum-doublet chemotherapies for patients with low or absent PD-L1 expression [36].

While the extent of tumour cell PD-L1 expression is critical to treatment selection, in patients whose 1L treatments do not follow guidelines for PD-L1 expression, many clinical factors such as comorbidities, performance status, or contraindications are considered when making the treatment decision. Patient preferences might also be relevant, in addition to factors such as progression-free survival, treatment delays, tumour-associated symptoms, treatment-related side effects, and out-of-pocket costs [37, 38].

Our analysis also demonstrated that QoL was impaired in patients with *EGFR*-WT/*ALK*-WT mNSCLC, including relative to normative reference values, and particularly for patients in France. Patients in France also differed from patients in other countries in terms of MCIDs for EQ-5D utility index and for FACT-Lung Cancer Subscale scores (excluding Italy). A real-world outcomes study of patients with mNSCLC who received IO or IO + chemotherapy showed that patient QoL (European Organization for the Research and Treatment of Cancer Quality of Life Questionnaire Core 30 [QLQ-30]) was similar between those on IO and IO + chemotherapy and not related to weeks on these treatments. Indirect comparison with clinical trial data showed that global QoL scores were worse than those 1L single-agent IO, alongside higher than expected symptom burden [39]. KEYNOTE-024 indicated improved QOL in patients prescribed pembrolizumab compared with platinum-doublet chemotherapy [40].

Additional to being the most common causes of cancer-related death worldwide [41], lung cancer is one of the most prevalent tumour types among patients with cancer who also have COVID-19 [42]. Patients with lung cancer are particularly vulnerable to COVID-19 infection, likely because abnormalities in their respiratory epithelium enable rapid entry of the virus into the lungs [42]. In this analysis of the impact of the COVID-19 pandemic, there were minimal differences in the demographics and clinical characteristics of patients with *EGFR*-WT/*ALK*-WT mNSCLC diagnosed in the pre-COVID-19 period and diagnosed during the COVID-19 period.Potential delays in diagnosis during the pandemic may be suggested by the greater difference between the frequency of a stage IVb and stage IVa diagnosis within the group diagnosed during COVID-19 than that found within the pre-COVID-19 group, although there was no apparent change in the tests and assessments used within the two diagnostic periods.

Nevertheless, COVID-19 had a substantial impact on patients’ management across European countries, with at least one area of management impacted for over one-third of all patients. The greatest impact appeared to be in the UK, where management was affected for approximately 80% of patients, specifically fewer consultations and tests/investigations, a move to video and telephone consultations from the usual face-to-face appointments, and prescribed treatment changes. These impacts could have been a result of the recommendations and strict guidelines of the National Health Service in the UK during the pandemic compared to the other countries. Patients in Germany appeared to be overall least impacted by the pandemic with regards to their mNSCLC treatment and management, particularly with minimal change in method of consultation and few treatment changes.

The differences in impact to patient management across Europe was likely to reflect the pressures that COVID-19 placed on health care systems and healthcare delivery. In the UK, COVID-19 has resulted in remote consultations becoming the new standard for patients with lung cancer, with this means of communication likely to remain a vital part of the diagnostic pathway [43]. In a Dutch survey, 30% of 2664 patients with cancer reported consequences for their oncological management, of which conversion from hospital visit to consultation by phone or video was most frequently reported [44]. Clinicians have had to balance the risk of delaying evaluation and management against those of exposing patients to COVID-19 in hospital settings and exposing healthcare professionals to asymptomatic patients. Moreover, the disruption from COVID-19 exposure and resource reallocation, as a result of the pandemic, have led to the development of new recommendations to replace current guidelines for clinicians managing patients with lung cancer such as delays in evaluation and treatment in specific cases [45].

Decision making in the treatment of patients with lung cancer during the COVID-19 pandemic has also presented challenges as to whether to offer, modify, postpone or cancel treatments [46]. While few changes to the prescribed treatment were observed, there seemed to be a small shift towards use of immunotherapy at 1L in those diagnosed during COVID-19 period from the pre-COVID period. Chemotherapy and immunotherapy have previously been reported to be the most frequently adjusted treatments during the pandemic [44].

Other factors may also contribute to the apparent changes in treatment patterns during the COVID-19 pandemic. Increased prescribing of immunotherapy may have been associated with recent advances in 1L immunotherapy. In November 2020, within the period used to define the COVID-19 cohort for this analysis, the European Medicines Agency approved nivolumab plus ipilimumab with two cycles of chemotherapy for 1L treatment of mNSCLC, in adults whose tumours have no sensitising EGFR mutation or ALK translocations [47]. Moreover, guidelines for treatment made during the COVID pandemic recommend priorities for patients with metastatic disease, including use of 1L chemotherapy, IO + chemotherapy, and IO to improve prognosis, cancer-related symptoms, and QoL [48]. Anti-PD-(L)1 scheduled treatment cycles may also be modified/delayed to reduce clinical visits. Findings from several registries indicate that patients treated with immunotherapy alone have equivalent or better outcomes than those receiving other cancer treatments [46]. As such, immunotherapy has mainly been continued, but with the use of longer cycle options where available, and chemotherapy-based regimens have been used only when necessary [43]. In addition, when it comes to the implementation of new therapeutic strategies such as immunotherapy there is usually a delay between reimbursement and regulatory approval which impacts the timing of real-world implementation of new strategies. This delay can yield unexpected results when it comes to characterisation of real-world treatment use in clinical practice, particularly in this case the use of immunotherapy. Together, these findings suggest that the increase in use of 1L immunotherapy in our study would have occurred regardless of the COVID-19 pandemic, due to evolving treatment landscape.

### Strengths and limitations

The DSP approach to collecting data has limitations, including its point-in-time design, which prevents any conclusions about causal relationships but allows for identification of significant associations. The DSP is not based on a true random sample of physicians; while minimal inclusion criteria governed the selection of the participating physicians, participation was influenced by willingness to complete the survey. Patients participating in the surveys may not reflect the general mNSCLC population, as patients who visit more frequently may be more severely affected, require more monitoring, treatment adjustments, or have more emergency visits than those who do not consult their physician as frequently. They also represent a pragmatic sample that may not be representative of the overall population of physicians treating NSCLC. Patient diagnosis and response to treatment was based on the judgement and diagnostic skills of the respondent physician, as there was no formalized diagnostic or response checklist, although this is entirely consistent with the decisions made by physicians in real-world clinical practice. Within the main sample, patients were recruited prospectively at the time of consultation, and the oversample was collected retrospectively. The quality of these data depends on the accurate reporting of information by physicians and patients, and therefore may be subject to recall bias, however data were collected at time of consultation and physicians had access to historical medical records, which is expected to reduce this potential for bias.

The impact of COVID-19 on diagnosis and treatment in 1L mNSCLC may not have been fully apparent at the point of data collection during the pandemic; there may be longer-term impacts of COVID-19 on diagnosis and treatment. However, it was possible to explore the initial impact of COVID-19 by comparing patients diagnosed pre-COVID-19 with those diagnosed during the COVID-19 when physicians and patients across Europe were in national ‘lockdown’. Our data look at specific time periods before and during the COVID-19 pandemic, and the findings are likely to change over time with the waves of infection within countries and as health systems adapted to operating with the disease.

## Conclusions

This analysis of the characteristics and the current diagnostic landscape in patients with *EGFR*-WT/*ALK*-WT mNSCLC across Europe found that IO as well as chemotherapy-based regimens were frequently prescribed as 1L treatment. The majority (~ 80%) of patients with PD-L1 expression of ≥ 50% were receiving 1L IO, which was used across all histological types investigated (squamous cell carcinoma, adenocarcinoma, large cell carcinoma). However, QoL of these patients was generally lower than normative reference values and variable across Europe, implying the need for more effective use of current treatments or novel therapies to manage patients with *EGFR*-WT/*ALK*-WT mNSCLC.

Investigating the impact of COVID-19 on the treatment of patients with *EGFR*-WT/*ALK*-WT mNSCLC, there was indication of delays in diagnosis during COVID-19, but with no apparent change in the tests and assessments used. Additionally, COVID-19 had a substantial impact on patients’ management across the five European countries. The UK was particularly affected in terms of consultations, tests/investigations, and prescribed treatment changes, while the impact on patients in Germany appeared to be relatively low. Few changes were made to prescribed treatments during COVID-19 but there was a small shift towards use of 1L immunotherapy potentially as a result of the changing therapeutic landscape.

Despite immunotherapy, current treatment for mNSCLC remains suboptimal, with response and sustained effectiveness in only the minority of patients [48]. Further investigation into characterisation of patients with mNSCLC is warranted, alongside its potential to guide treatment choice, and all novel potentially effective immune therapies for mNSCLC should be evaluated, particularly with the advent of the COVID-19 pandemic.

## Supplementary Information

**Additional file 1.**

## Data Availability

All data, i.e., methodology, materials, data and data analysis, that support the findings of this survey are the intellectual property of Adelphi Real World. All requests for access should be addressed directly to Hollie Bailey at hollie.bailey@adelphigroup.com.
