# Supplementary material for: Treatment patterns and outcomes of patients with metastatic non-small cell lung cancer in five European countries: a real-world evidence survey
Source: BMC Cancer. 2023 Jun 30;23:603. doi: 10.1186/s12885-023-11074-z (PMC10311888; doi:10.1186/s12885-023-11074-z)
Supplement: Supplementary file 1 — Additional file 1. [file 12885_2023_11074_MOESM1_ESM.docx]

**Supplementary Table 1**. Tests and assessments used in diagnosis and monitoring status of *EGFR*-WT/*ALK*-WT mNSCLC

|  | **Total population** | **1L treatment** | **p value** |
| --- | --- | --- | --- |
| Tests/assessment, n (%) |  |  |  |
| Used in NSCLC diagnosis |  |  |  |
| n | 1073 | 915 |  |
| Biopsy | 955 (89.0) | 815 (89.1) | 0.5471 |
| Blood test | 920 (85.7) | 788 (86.1) | 0.2691 |
| CT scan-chest | 897 (83.6) | 764 (83.5) | 0.6773 |
| FDG-PET scan | 619 (57.7) | 520 (56.8) | 0.0837 |
| Bronchoscopy | 604 (56.3) | 518 (56.6) | 0.6375 |
| X-ray | 565 (52.7) | 492 (53.8) | 0.1604 |
| Pulmonary function test | 506 (47.2) | 442 (48.3) | 0.1009 |
| Radioisotope/bone scan | 271 (25.3) | 226 (24.7) | 0.4731 |
| MRI | 251 (23.4) | 198 (21.6) | 0.0024 |
| Ultrasound | 171 (15.9) | 148 (16.2) | 1 |
| Used in monitoring |  |  |  |
| n | 1071 | 913 |  |
| Blood test | 911 (85.1) | 776 (85.0) | 0.5162 |
| CT scan-chest | 575 (53.7) | 455 (49.8) | <0.0001 |
| X-ray | 272 (25.4) | 224 (24.5) | 0.0182 |
| FDG-PET scan | 194 (18.1) | 143 (15.7) | <0.0001 |
| Pulmonary function test | 111 (10.4) | 94 (10.3) | 0.7032 |
| MRI | 90 (8.4) | 52 (5.7) | <0.0001 |
| Ultrasound | 92 (8.6) | 72 (7.9) | 0.0752 |
| Radioisotope/bone scan | 82 (7.7) | 56 (6.1) | <0.0001 |
| Biopsy | 72 (6.7) | 57 (6.2) | 0.003 |
| Bronchoscopy | 60 (5.6) | 51 (5.6) | 1 |

ALK, anaplastic lymphoma kinase; CT, computerized tomography; EGFR, epidermal growth factor receptor; *EGFR*-WT/*ALK*-WT, i.e., no sensitising EGFR mutation or ALK translocation; wild type; FDG PET, fluorodeoxyglucose-positron emission tomography; MRI, magnetic resonance imaging; mNSCLC, metastatic non-small cell lung cancer; 1L, first line.
